# The Voluntary Hospitals' Budget

**Published:** 1919-06-21

**Authors:** 


### THE VOLUNTARY HOSPITALS' BUDGET.

At the close of the Great War the voluntary hospitals find themselves called upon to continue under conditions which, in a few years, have changed to an extent that could hardly have been foreseen. Although statesmen have given assurrances that the voluntary system, so dear to the public mind, will continue, it is clear that the trend of legislation, and the needs of the sick, will call for more extensive work on the part of the hospitals. Whether it be in the direction of pre-natal clinics, infant welfare, the more thorough treatment of venereal diseases, of consumption or other scourges, it cannot be doubted that the responsibilities of our great medical charities have increased. The question of finance becomes more and more important; State subsidy, unaccompanied by undue State interference, may help to solve that part of the question, but few would wish that the useful outlet for an expression of public sympathy with the sick and the suffering should be removed.

On Hospital Sunday, June 22, 1919, the call for a renewal of that practical sympathy is more than ever necessary, and of this, throughout the mighty struggle, the hospitals have proved themselves most worthy. No part of our unpreparedness was more evident at the beginning of the war than the arrangements for the treatment of sick and wounded sailors and soldiers, and to meet this urgent need the hospitals freely offered their wards and their trained workers in addition to sending to our far-flung battle-lines the doctors and nurses who had gained their skilful knowledge within the walls of our great hospitals.

Before the war the ordinary expenditure of the Metropolitan Voluntary Hospitals and Dispensaries usually increased by about £40,000 a year. In 1913 it was about £1,295,000, in 1914 about £1,334,000, in 1915 it rose to £1,400,000, in 1916 to about £1,600,000, and since it has been rising appreciably. To meet this heavy expenditure the hospitals and dispensaries have to rely upon three main sources of revenue.

First and most important of these are the gifts of the charitable, for which we are now appealing, such as subscriptions, donations, entertainments, grants raised by King Edward's Hospital Fund and the Hospital Sunday and Saturday Funds, and other receipts of a voluntary character. This source of revenue, together with the payments made by patients (including grants for the maintenance of discharged sailors and soldiers), supplies about two-thirds of the amount required for the upkeep of the institutions, and may be taken as worth about £1,300,000 a year. But to reach that figure a renewal of the generosity of friends in the past is necessary, for new friends but take the place of those who are lost by death or changed circumstances. Expenses are increasing by leaps and bounds, and it is improbable that they will be less than £2,000,000, and the £1,300,000 above mentioned would bring in exactly 13s. of every £1 required. Secondly, there is the income from investments and property. The value of this, because of higher rates of interest, has increased and should continue to do so. The amount received in 1916 was £382,000, in 1917, £414,000, and the figure for 1918 will be, perhaps, £420,000, or equal to four shillings towards every sovereign required.

These two fairly stable sources of income may be expected to provide 16s. in the pound, but the other 4s., or about one-fifth of the total requirements, must be looked for as coming from a more precarious source—indeed, the only remaining one from which revenue can be derived—viz., the gifts left to hospitals in the shape of legacies. These vary in amount from year to year; in a period of ten years they have fallen as low as £240,000, and reached as high as £451,000, the last figure being for 1914. The figure for 1917 proves to be about the same as that for 1916. But in any case legacies should be regarded as extraordinary income, and should be utilised to assist in meeting the expenditure required for rebuilding, for improvements requisite to keep pace with the rapid march of science, or for extension where overcrowding has become apparent, all of which items of extraordinary expenditure have now to be met by special appeals to those who already have given largely.

But extraordinary expenditure finds no place in the present budget. Ordinarily about a quarter of a million a year is spent in this way, but most schemes of rebuilding, enlargement, or alterations have been postponed.

In conclusion, having shown that such support is necessary, may we ask the old supporters of the Metropolitan Hospitals and Dispensaries to give this year, as nearly as possible, as liberally as they have done in the past? And we call upon those who have not hitherto given to remember what the voluntary hospitals have done, and are doing for the outside community, to recognise under what difficulties they are working at present, and to make special and generous gifts on Hospital Sunday.

### INCOME.

Table Showing Income of the Metropolitan Hospitals and Dispensaries in the Ten Years 1908 to 1917.

| Year | From the Living | | From the Dead | | | | Total Income |
|---|---|---|---|---|---|---|---|
| | Subscriptions, Donations, Patients' Payments, etc. | Percentage of Total | Investments | Percentage of Total | Legacies | Percentage of Total | |
| | £ | | | | £ | | £ |
| 1908 | 712.461* | 56 | 290,491 | 23 | 265,493 | 21 | 1,268,445 |
| 1909 | 701,458 | 57 | 294,206 | 24 | 240 523 | 19 | 1,236,187 |
| 1910 | 703,841 | 50 | 309.320 | 22 | 394.478 | 28 | 1,407,639 |
| 1911 | 738,622 | 53 | 329,253 | 24 | 322.949 | 23 | 1,390,824 |
| 1912 | 741,439 | 55 | 338,425 | 25 | 272,090 | 20 | 1,351,954 |
| 1913 | 845,908* | 52 | 349,295 | 22 | 424 339 | 26 | 1,619,542 |
| 1914 | 801 239 | 49 | 366,841 | 23 | 451,569 | 28 | 1,619,649 |
| 1915 | 978,294 | 62 | 368,536 | 23 | 242 049 | 15 | 1,588 879 |
| 1916 | 1,143.417 | 63 | 382,377 | 21 | 301,282 | 16 | 1.827.076 |
| 1917 | 1,300,539 | 65 | 414,636 | 21 | 290,025 | 14 | 2,005,200 |

* Includes £72,565 and £103,970, the amounts received by the London Hospital in 1908 and 1913 respectively as the result of its Quinquennial Appeals in those years.

